# Pseudocirrhosis and portal hypertension in patients with metastatic cancers: a systematic review and meta-analysis

**DOI:** 10.1038/s41598-022-24241-2

**Published:** 2022-11-18

**Authors:** Rosanna Villani, Francesca Di Cosimo, Moris Sangineto, Antonino Davide Romano, Gaetano Serviddio

**Affiliations:** Liver Unit, C.U.R.E. (University Centre for Liver Disease Research and Treatment), Department of Medical and Surgical Sciences, University of Foggia, Viale Pinto 1, 71122 Foggia, Italy

**Keywords:** Liver, Cancer epidemiology

## Abstract

Pseudocirrhosis is a clinical and radiological entity mimicking liver cirrhosis in patients without a history of chronic liver disease. We performed a systematic review and meta-analysis of the current literature to evaluate the state-of-the-art and investigate the epidemiology and clinical features of pseudocirrhosis. We searched PubMed, Web of Science and Scopus for literature published until February 28, 2022. We included in the final analysis 62 articles (N = 389 patients): 51 case reports (N = 64 patients), 5 case series (N = 35 patients) and 6 observational studies (N = 290 patients). About 80% of patients included in the case reports and case series had breast cancer. Most patients had at least one clinical sign of portal hypertension and ascites was the most common clinical manifestation of portal hypertension. The median time from pseudocirrhosis to death was 2 months (IQR 1–7 months). Alkylating agents and antimitotics were the most common classes of anticancer drugs reported in our study population. Notably, about 70% of patients received three or more anticancer drugs. Finally, pseudocirrhosis is a condition that occurs in patients with hepatic metastases and may have a negative impact on survival and clinical management of patients because of the potential development of portal hypertension and its complications.

## Introduction

Pseudocirrhosis is a rare but challenging condition mimicking radiographically and clinically liver cirrhosis without the typical histopathological changes observed in cirrhosis^1^. A condition referred to as *hepar lobatum carcinomatosum* was described for the first time in 1924 by Busni et al. who observed irregular and lobulated hepatic contours in their autopsy report of a 37-year-old woman with breast cancer^2^.

Over the next decades, several authors have observed this phenomenon in the metastatic setting and described multifocal scars and compensatory hyperplasia in spared liver tissue of patients with *hepar lobatum carcinomatosum*^3–6^ but only in 1994, Young et al., due to its clinical and radiological features renamed this condition as “pseudocirrhosis”^7^.

The prevalence of pseudocirrhosis is not known and data on its clinical features, management and prognosis are limited because available information has been obtained mostly from case reports and small observational studies^8^ which reported cirrhosis-like changes in patients with liver metastases or without liver metastases after systemic chemotherapy^9^.

The mechanism underlying the disease development is unclear, however liver biopsies obtained from patients with pseudocirrhosis have shown desmoplastic fibrosis, nodular regenerative hyperplasia, or diffuse infiltration of tumor cells^10,11^.

Currently, there are no criteria for the diagnosis, which is based mainly on imaging techniques and, currently, the term pseudocirrhosis includes a large spectrum of pathophysiological mechanisms.

Based on the available studies, two main mechanisms could be involved in pseudocirrhosis development: toxicity of systemic therapy and changes after malignant liver infiltration^12^.

Early diagnosis and appropriate monitoring are particularly relevant in patients with pseudocirrhosis because clinical manifestations of portal hypertension, such as variceal bleeding or hepatic encephalopathy, may occur^8^.

We performed a systematic review and meta-analysis of the current literature to evaluate the state-of-the-art and investigate the epidemiology and clinical features of pseudocirrhosis, cancers and drugs associated with pseudocirrhosis development.

## Materials and methods

We conducted a systematic review of the literature and meta-analysis of case reports, case series and observational studies in accordance with the preferred reporting items for systematic review and meta-analyses (PRISMA) guidelines^13^.

### Data sources and searches

We searched PubMed, Web of Science and Scopus for literature published until February 28, 2022.

A systematic search using “pseudocirrhosis” OR “hepar lobatum carcinomatosum” OR “hepar lobatum” as keywords was performed. Relevant citations were retrieved after screening full-text articles.

We also checked the reference lists of the included articles and review articles identified by the electronic databases. Literature searches were conducted without language or data restrictions. The review protocol was not recorded.

### Study selection

We included in our analysis all case reports, case series and observational studies reporting data on.

1. the prevalence and clinical manifestations of pseudocirrhosis
2. overall survival in patients with pseudocirrhosis, time from liver metastasis diagnosis to pseudocirrhosis, time from pseudocirrhosis diagnosis to death
3. anticancer protocols used in patients with pseudocirrhosis
4. prevalence of portal hypertension and its complications in patients with pseudocirrhosis

We excluded from our analysis manuscript reporting data on pseudocirrhosis in patients < 18 years old.

Search strategies were implemented by using the reference management software EndNote^®^ (version 20, Clarivate Analytics, Philadelphia, PA, USA).

After the removal of duplicates, the remaining full-text articles were assessed for inclusion.

All publications including data on the clinical course of less than four individuals were considered as case reports, whereas articles reporting 4 or more clinical cases were classified as case series^14^.

The selection of articles based on the criteria described above was performed independently by two of the authors (RV and FDC), and conflicts were resolved by a third investigator (GS).

Data extracted included the country of origin of the published cases, year of publication, patient demographics, underlying conditions/co-morbidities, clinical manifestations, blood tests and drugs used to treat cancer. In case of overlapping population, only manuscripts reporting the most updated data were included in the final analysis.

### Quality assessment

The quality of observational studies included in our analysis was evaluated by using the Newcastle–Ottawa Scale (NOS). The NOS includes three domains: selection, comparability and outcome. It classified the risk of bias as low (7–9 stars; high quality), moderate (4–6 stars; fair quality) and high (1–3 stars; low quality)^15^.

The quality of case reports and case series was evaluated according to the “tool for evaluating the methodological quality of case reports and case series” proposed by Murad and colleagues, based on selection, ascertainment, causality, and reporting domains^16^.

More specifically, the checklist assesses for each case report or case series the availability of data from the whole experience of the investigator (centre), the selection method, clear report of outcome and ascertainment of exposure; details on differential diagnosis; occurrence of challenge/rechallenge phenomenon; dose–response effect description; sufficient length of follow-up; report of sufficient details to allow other investigators to replicate the research or to allow practitioners make inferences related to their own practice.

### Statistical analysis

Data analysis was conducted using STATA (StataCorp. 2015. Stata Statistical Software: Release 14. College Station, TX: StataCorp LP) and Graphpad Prism (version 8.0.0, Graphpad Software, San Diego, California USA).

Patients’ characteristics at baseline were summarized descriptively. Data on overall survival, time from liver metastasis diagnosis to pseudocirrhosis, time from pseudocirrhosis diagnosis to death were analyzed and reported as median and interquartile range (IQR). Overall survival was defined as the length of time from cancer diagnosis or the start of anticancer treatment to death. Summary data on drug use are reported as frequencies. Proportional meta-analysis of varices, splenomegaly and ascites prevalence rates was conducted using Stata module Metaprop^17^. The overall estimates were calculated using random effect models. Heterogeneity between studies was evaluated using the *I*^2^ statistic with a cut-off point of ≥ 50% and a P value < 0.10 on the χ^2^ test was defined as a significant degree of heterogeneity.

Kappa coefficient was used to explore the between-reviewer agreement in terms of decision to include studies^18^.

Sensitivity analysis was performed to assess whether results were robust enough to potentially influence decision-making. One or more studies were excluded to assess whether exclusions significantly change the estimation of the pooled prevalence.

## Results

### Study selection and patient population in case reports/case series and observational studies

We included in the final analysis 62 articles (N = 389 patients):

- 51 case reports (N = 64 patients),
- 5 case series (N = 35 patients)
- 6 observational studies (N = 290 patients).

Between- reviewer agreement for the study selection assessed by the kappa coefficient was 0.94 (95%CI 0.87–0.99).

**Figure 1:**
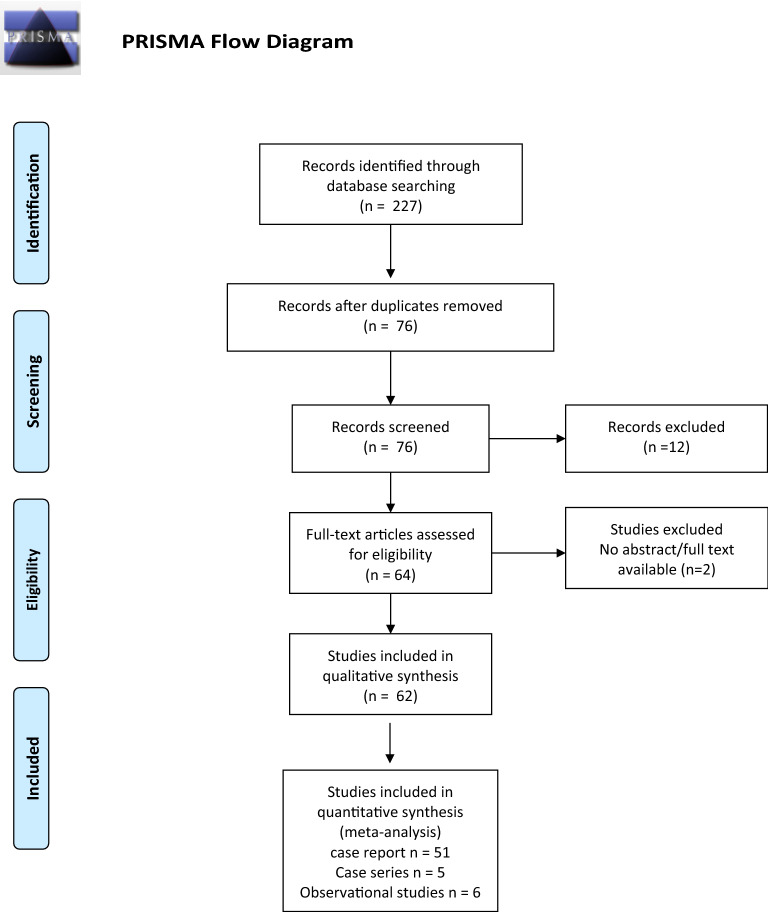
PRISMA flowchart of selected studies.

Study characteristics and the PRISMA flowchart are shown in Tables 1, 2, 3 and Fig. 1. Most article were from North and South America (N = 25; 40.3%), followed by Europe (N = 17; 27.4%) and Asia (N = 16; 25.8%). The country was not available for four publications (6.5%) (Table 1).

**Table 1:** Case reports included in the final analysis.

Author	Country	N = 64	Malignancy	Age (years)	Gender	Liver histology	Platelets (n°/mm^3^)	Total bilirubin (mg/dl)	Albumin (g/dl)	Chemotherapy
Adler et al.^33^	UK	1	Breast	63	F	Breast cancer cells	198.000	2.92	3.5	Yes
Battisti et al.^34^	Italy	1	Colon	69	M	n.a.	n.a.	n.a.	n.a.	Yes
Borja et al.^35^	US	1	Breast	46	F	Breast cancer cells; fibrosis	225.000	6.8	2.7	Yes
Busni et al.^2^	US	1	Breast	37	F	Breast cancer cells; fibrosis	n.a.	n.a.	n.a.	No
Cerny et al.^36^	Czech Republic	1	Breast	68	F	Breast cancer cells; fibrosis	n.a.	n.a.	n.a.	Yes
Cervoni et al.^37^	France	1	Breast	52	F	Breast cancer cells; fibrosis	n.a.	2.92	n.a.	Yes
Chandrakan et al.^38^	US	1	Breast	45	F	Breast cancer cells; fibrosis	n.a.	n.a.	n.a.	Yes
Chin et al.^39^	n.a.	1	Stomach	63	n.a.	n.a.	n.a.	n.a.	n.a.	Yes
Deprez et al.^40^	Belgium	1	Breast	59	F	Breast cancer cells; fibrosis	n.a.	n.a.	n.a.	Yes
Eidenschink et al.^41^	US	1	Breast	83	F	Breast cancer cells	n.a.	7.9	2.6	No
Finocchi et al.^42^	Italy	1	Breast	46	F	Breast cancer cells; fibrosis	n.a.	3.3	2.6	No
Fournier et al.^43^	US	1	Breast	52	F	Breast cancer cells	50.000	17	n.a.	Yes
Graber et al.^3^	France	1	Breast	57	F	Breast cancer cells; fibrosis	70.000	3.45	n.a.	No
Harry et al.^44^	US	1	Thyroid	49	F	Medullary thyroid cancer cells; fibrosis	n.a.	n.a.	3.1	Yes
Hidalgo-Blanco et al.^45^	Spain	1	Breast	39	F	Breast cancer cells	n.a.	n.a.	n.a.	Yes
Honma et al.^46^	Japan	1	Breast	48	F	Breast cancer cells; fibrosis	n.a.	n.a.	n.a.	Yes
Jungst et al.^47^	Germany	1	Breast	70	F	Breast cancer cells; fibrosis	98.000	3.5	1.8	n.a.
Kang et al.^22^	US	1	Pancreas	55	F	n.a.	n.a.	1.54	1.7	Yes
Kashyap et al.^48^	India	1	Breast	35	F	n.a.	n.a.	n.a.	n.a.	Yes
Kears et al.^49^	n.a.	1	Breast	43	F	n.a.	n.a.	n.a.	n.a.	n.a.
Klinge et al.^6^	Germany	1	Breast	n.a.	n.a.	n.a.	n.a.	n.a.	n.a.	n.a.
Kobashigawa et al.^50^	Japan	1	Esophagus	68	M	n.a.	n.a.	n.a.	n.a.	Yes
Lee et al.^8^	Korea	1	Breast	47	F	Breast cancer cells	n.a.	n.a.	n.a.	Yes
Leyden et al.^51^	Ireland	1	Breast	n.a.	F	Breast cancer cells	95.000	2.87	3	n.a.
Liu et al.^52^	Taiwan	1	Breast	44	F	n.a.	n.a.	11.8	n.a.	Yes
Marzuk et al.^53^	US	1	Breast	63	F	Breast cancer cells	121.000	1.45	2.5	Yes
Maynard et al.^54^	US	1	Breast	52	F	Breast cancer cells; fibrosis	n.a.	5.2	2.4	no
Mitani et al.^55^	Japan	1	Stomach	74	M	Adenocarcinoma	n.a.	2.8	1.8	Yes
Nakajima et al.^56^	Japan	1	Breast	68	F	Breast cancer cells; fibrosis	93.000	4.97	3.1	Yes
Ojeda et al.^57^	n.a.	1	Lung	n.a.	n.a.	Oat cell bronchial carcinoma	n.a.	n.a.	n.a.	n.a.
Patel et al.^58^	US	1	Breast	80	F	n.a.	n.a.	8.9	2.1	Yes
Sass et al.^9^	US	1	Breast	55	F	Breast cancer cells; fibrosis	n.a.	6.8	2.4	Yes
Tambe et al.^59^	US	1	Breast	71	F	Breast cancer cells	76.000	0.4	n.a.	n.a.
Teke et al.^60^	Turkey	1	Colon	50	M	Adenocarcinoma	n.a.	n.a.	n.a.	Yes
Uhlmann et al.^61^	Germany	1	Breast	59	F	Breast cancer cells	n.a.	n.a.	n.a.	n.a.
Wallace et al.^62^	US	1	Breast	42	F	Breast cancer cells	n.a.	n.a.	n.a.	Yes
Zanazanian et al.^63^	US	1	Ovary	43	F	Ovarian carcinoma	n.a.	n.a.	n.a.	Yes
Zeina et al.^64^	Israel	1	Breast	82	F	n.a.	n.a.	n.a.	n.a.	Yes
Aoyagi et al.^65^	Japan	3	Breast	65	F	n.a.	181.000	1.1	3.1	Yes
				65	F	n.a.	246.000	0.8	4.3	
				68	F	n.a.	414.000	0.8	2.2	
Jeong et al.^25^	Korea	2	Breast	53	F	n.a.	214.000	4.3	3.1	Yes
				25	F	n.a.	50.000	2.4	2.7	
Nascimento et al.^11^	Brazil	2	Breast	62	F	Breast cancer cells fibrosis	n.a.	3.2	n.a.	Yes
				46	F	Breast cancer cells fibrosis	27.000	7.2	n.a.	
Gravel et al.^5^	Canada	2	Breast	42	F	Breast cancer cells fibrosis	n.a.	1.46	n.a.	Yes
				58	F	Breast cancer cells fibrosis	n.a.	27.1	n.a.	
Qizilbash et al.^66^	n.a.	3	Breast	50	n.a.	n.a.	n.a.	n.a.	n.a.	Yes
				46	n.a.	n.a.	n.a.	n.a.	n.a.	
				70	n.a.	n.a.	n.a.	n.a.	n.a.	
Vuppalanchi et al.^67^	US	2	Breast	47	F	Breast cancer cells fibrosis	n.a.	2	n.a.	Yes
				61	F	n.a.	n.a.	2.2	n.a.	
Geeroms et al.^68^	Belgium	3	Breast	56	F	Breast cancer cells fibrosis	72.000	0.9	n.a.	Yes
				70	F	Nodular regenerative hyperplasia	141.000	n.a.	n.a.	
				79	F	Breast cancer cells	250.000	n.a.	n.a.	
Mizuyama et al.^69^	Japan	2	Breast	n.a.	n.a.	n.a.	n.a.	n.a.	n.a.	n.a.
Shirkoda et al.^70^	US	3	Breast	n.a.	n.a.	n.a.	n.a.	n.a.	n.a.	n.a.
Shinoda et al.^23^	Japan	1	Stomach	72	M	n.a.	n.a.	1.8	3.5	Yes
Shijubou et al.^71^	Japan	1	Lung	50	M	Lung adenocarcinoma fibrosis	n.a.	n.a.	n.a.	Yes
Nakano et al.^72^	Japan	1	Lung	64	M	Lung adenocarcinoma	n.a.	n.a.	2.7	Yes
Basinger et al.^73^	US	1	Stomach	71	M	Gastric adenocarcinoma; fibrosis; nodule	n.a.	n.a.	n.a.	Yes

**Table 2:** Case series included in the final analysis.

Author	Country	N = 35	Malignancy	Age	Gender	Liver biopsy	Platelets (n°/mm^3^)	Total bilirubin (mg/dl)	Albumin (g/dl)	Chemotherapy
Adike et al.^74^	US	6	Breast	46	F	n.a.	135.000	1.1	2.6	6/6 (100%)
				65	F	n.a.	116.000	0.6	3.3	
				66	F	n.a.	n.a.	4.3	n.a.	
				60	F	n.a.	90.000	8.7	2.2	
				76	F	n.a.	134.000	0.3	3.7	
				64	F	Breast cancer cells	329.000	2	3.2	
Sonnenblick et al.^10^	Israel	5	Breast	37	F	n.a.	n.a.	n.a.	n.a.	5/5 (100%)
				43	F	n.a.	n.a.	n.a.	n.a.	
				51	F	n.a.	n.a.	n.a.	n.a.	
				33	F	n.a.	n.a.	n.a.	n.a.	
				53	F	n.a.	n.a.	n.a.	n.a.	
Alberti et al.^4^	France	5	Breast	Mean 61 (range 56–69)	F	n.a.	n.a.	2.3	n.a.	5/5 (100%)
					F	n.a.	n.a.	2.1	n.a.	
					F	n.a.	n.a.	2.2	n.a.	
					F	Nodular regenerative hyperplasia	n.a.	2.6	n.a.	
					F	Nodular regenerative hyperplasia	n.a.	2.2	n.a.	
Gomez Raposo et al.^75^	Spain	10	Breast	Mean 57 (range 47–73)	F	n.a.	n.a.	n.a.	n.a.	10/10 (100%)
Shreve et al**.**^76^	US	9	Colon (n = 5) NET (n = 3) Liver (n = 1)	61.2 ± 9.5	4 F 5 M	7 out of 9 patients Cancer cells	n.a.	n.a.	n.a.	7/9 (77.8%)

**Table 3:** Observational studies included in the final analysis.

Author	Year	Country	N = 290	Malignancy	Age	Liver biopsy	Platelets (n°/mm^3^)	Total bilirubin (mg/dl)	Albumin (g/dl)	Chemotherapy
Oliai et al.^1^	2019	US	37	Breast	49.3 SD 13.5	n.a.	n.a.	6 SD 6.7	2.7 SD 0.7	37/37 (100%)
Qayyum et al.^21^	2007	US	68	Breast	n.a.	n.a.	n.a.	n.a.	n.a.	68/68 (100%)
Fennessy et al.^19^	2004	Switzerland	29	Breast	n.a.	n.a.	n.a.	n.a.	n.a.	29/29 (100%)
Young et al.^7^	1994	US	22	Breast	48	nodular regenerative hyperplasia; breast cancer cells	n.a.	n.a.	n.a.	22/22 (100%)
Gopalakrishnan et al.^20^	2018	US	86	Breast	57.5 (range 32.4–82.4)	n.a.	Median 195.000 IQR 137.000–217.000	Median 0.5 IQR 0.3–0.9	Median 3.6 IQR 3.2–4	86/86 (100%)
Engelman et al.^12^	2020	Belgium	48	Breast	50.6 SD 11.6	n.a.	187.000 SD 82.7	1 SD 1	3.5 SD 0.8	48/48 (100%)

Underlying liver diseases were not observed in all articles included in the final analysis, except for one study, which included 2 patients with alcohol consumption > 20 g/daily^12^.

In patients population obtained from case reports and case series, data on previous anticancer treatment were not available for 9 patients (9.1%).

Among cases published with a complete history of cancer treatment, most patients (83 of 90; 92.2%) who developed pseudocirrhosis had a history of anticancer treatment, whereas only 7 patients (7.8%) did not receive any chemotherapy before pseudocirrhosis diagnosis.

This last subgroup of patients received no treatment (5 patients), surgical treatment (1 patient) or orthotopic liver transplantation (1 patient with malignant hepatic hemangioendothelioma).

All patients included in the observational studies received anticancer treatment (290 of 290; 100%).

### Quality assessment

We evaluated the methodological quality of included case series and case reports according to the Murad’s checklist^16^ (Supplementary Tables S1, S2). Items addressing the challenge/rechallenge and the dose response effect were not applicable.

Quality of observational studies assessed by Newcastle–Ottawa Scale is reported in Supplementary Table S3.

### Cancers and risk of pseudocirrhosis—case reports/case series

Most patients affected by pseudocirrhosis and reported in case reports and case series had breast cancer (77 of 99 patients; 77.8%) whereas the remaining patients reported one of the following cancers: colon (N = 7; 7%), thyroid (N = 1; 1%), esophagus (N = 1; 1%), pancreas (N = 1; 1%), stomach (N = 4; 4%), ovarian carcinoma (N = 1; 1%) and lung carcinoma (N = 3; 3%), neuroendocrine (N = 3; 3%), hepatic hemangioendotelioma (N = 1; 1%) (Tables 1, 2).

Data on estrogen receptor and progesteron receptor were available in 26 and 23 patients with breast cancer, respectively. ER+ (estrogen-receptor positive) was observed in 23 of 26 patients whereas progesteron-receptor positive (PR+) was found in 14 of 23 patients. HER2 status was available for 22 patients and 59% of them (13 of 22 patients) were positive. No patient had triple-negative breast cancer.

Table 4 shows the histological subtypes of breast cancers in patients reported in case reports and case series. Most patients (74.4%) with breast cancer and pseudocirrhosis had an invasive ductal carcinoma followed by invasive lobular carcinoma (7%).

**Table 4:** Histological classification in patients with breast cancer and pseudocirrhosis reported in case report and case series.

Histological subtypes (N = 43)	Prevalence
Invasive ductal carcinoma	32 (74.4%)
Invasive lobular carcinoma	3 (7%)
Ductal carcinoma in situ	1 (2.3%)
Mixed ductal-lobular carcinoma	2 (4.6%)
Undifferentiated adenocarcinoma	1 (2.3%)
Unspecified breast cancer	4 (9.3%)

### Cancers and risk of pseudocirrhosis—observational studies

All patients included in the observational studies (N = 290 patients) had breast cancer. ER/PR expression patterns were reported in 2 out of 6 studies^19,20^ whereas two studies reported overall HR profile without data by subgroups^1,12^.

In particular, Fennessy et al. (N = 29 patients) reported an ER+ prevalence of 55.2% whereas Gopalakrishnan et al. (N = 86 patients) found ER+ in 83.7% of the study population^19,20^. However, the prevalence of PR+ was 48.3% and 58.1% respectively. Four out of six studies reported data on the HER status of enrolled patients^1,12,19,20^. The prevalence of HER-2-positive breast cancers ranges between 17.4% reported by Gopalakrishnan et al.^20^ and 41.4% by Fennessy et al.^19^.

Only one study reported the histological subtypes of breast cancer for enrolled patients^12^. The ductal carcinoma was the most common subtype (72.7%) observed among patients with pseudocirrhosis followed by lobular type (22.7%).

### Diagnosis of pseudocirrhosis—case reports/case series

Among patients reported in case reports and case series, only 49 patients underwent liver biopsy. In almost all cases (46 of 49 patients; 93.9%), liver biopsy revealed hepatic diffuse infiltration by tumor cells whereas three patients were diagnosed with nodular regenerative hyperplasia (Table 1).

In 69.7% of cases (69 of 99 patients) diagnosis was performed by imaging techniques (CT scan and/or MRI). CT scan was the only technique used in 49 of 99 patients, MRI in 3 of 99 cases; both CT scan and MRI in 8 cases. Finally, 9 patients observed by Shreve et al. were diagnosed with pseudocirrhosis using CT scan or MRI, but the author did not report the specific technique used for every single patient.

In 10 of 99 patients (10.1%) the diagnosis was performed or confirmed after autopsy.

### Diagnosis of pseudocirrhosis—observational studies

In observational studies (N = 6), there were no specific criteria for the diagnosis which was based mainly on imaging techniques.

Five of six studies^1,7,12,19,21^ reported information on imaging techniques used for the diagnosis, however only four articles reported radiological criteria^1,7,19,21^.

The pooled data from these studies showed that the diagnosis was performed mostly using CT scan. In two cases reported by Young et al. the diagnosis was obtained using CT and autopsy^7^. Radiologic criteria for the diagnosis of pseudocirrhosis reported by observational studies are summarized in Table 5.

**Table 5:** Radiologic criteria used for the diagnosis of pseudocirrhosis reported by observational studies.

Study	Imaging technique	Criteria
Oliai et al.^1^	CT scan	Hepatic capsular retraction Signs of portal hypertension
Qayyum et al.^21^	CT scan	Hepatic contour abnormalities Volume loss Caudate hypertrophy Hepatic enlargement Signs of portal hypertension
Fennessy et al.^19^	CT scan	Hepatic capsular retraction
Young et al.^7^	CT scan Autopsy	Lobular contour of the liver Lobar or segmental volume loss Enlargement of the caudate lobe
Engelman et al.^12^	CT scan MRI	Liver contour abnormalities
Gopalakrishnan et al.^20^	CT scan MRI Ultrasound	Not reported

### Portal hypertension in patients with pseudocirrhosis—case reports and case series

Portal hypertension was observed in about 80% of patients with pseudocirrhosis. Data on specific clinical manifestations of portal hypertension are reported in Table 6. Ascites and esophageal varices were the most common manifestations of portal hypertension. Particularly, ascites was reported in about half of the case reports or case series, whereas esophageal varices were found in about one-third of patients. However, encephalopathy was the less common clinical manifestation of portal hypertension with a prevalence of only 9.1% of patients included in the case reports and case series.

**Table 6:** Clinical manifestations of portal hypertension reported in case reports/case series and observational study.

Portal hypertension	Case reports and case series (N = 99)
**Present**	79 (79.8%)
Ascites	43 (43.4%)
Esophageal varices	24 (24.2%)
Gastrointestinal bleeding	14 (14.1%)
Portal vein thrombosis	5 (5%)
Splenomegaly	15 (15%)
Encephalopathy	9 (9.1%)
**Absent or not reported**	20 (20.2%)

### Portal hypertension in patients with pseudocirrhosis—observational studies

**Figure 2:**
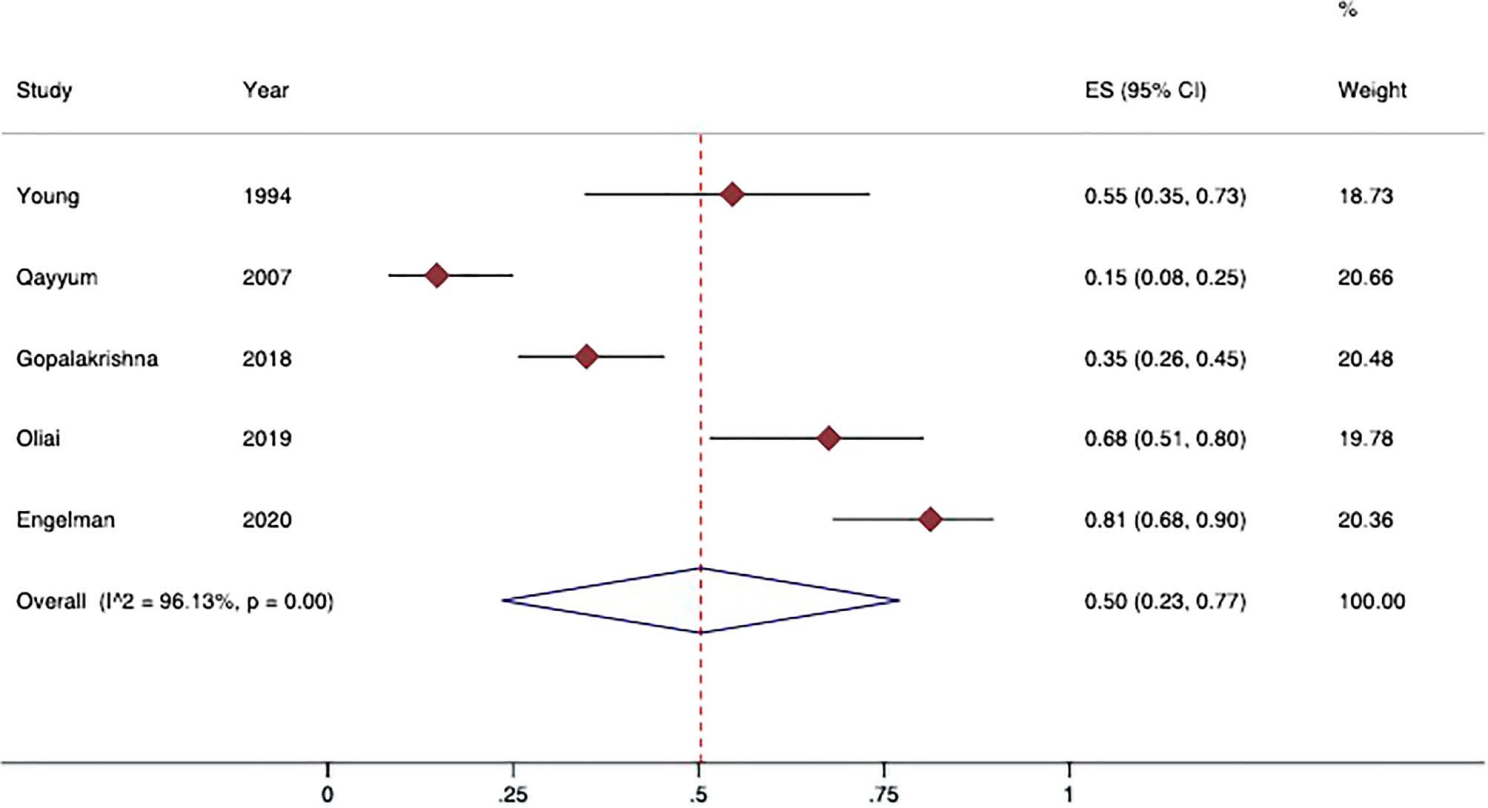
Prevalence of ascites in patients with pseudocirrhosis. Forest plot of overall pooled prevalence of ascites (random effect model); data from observational studies. 95%CI: 95% Confidence Intervals; ES: effect size; I^2^: heterogeneity.

**Figure 3:**
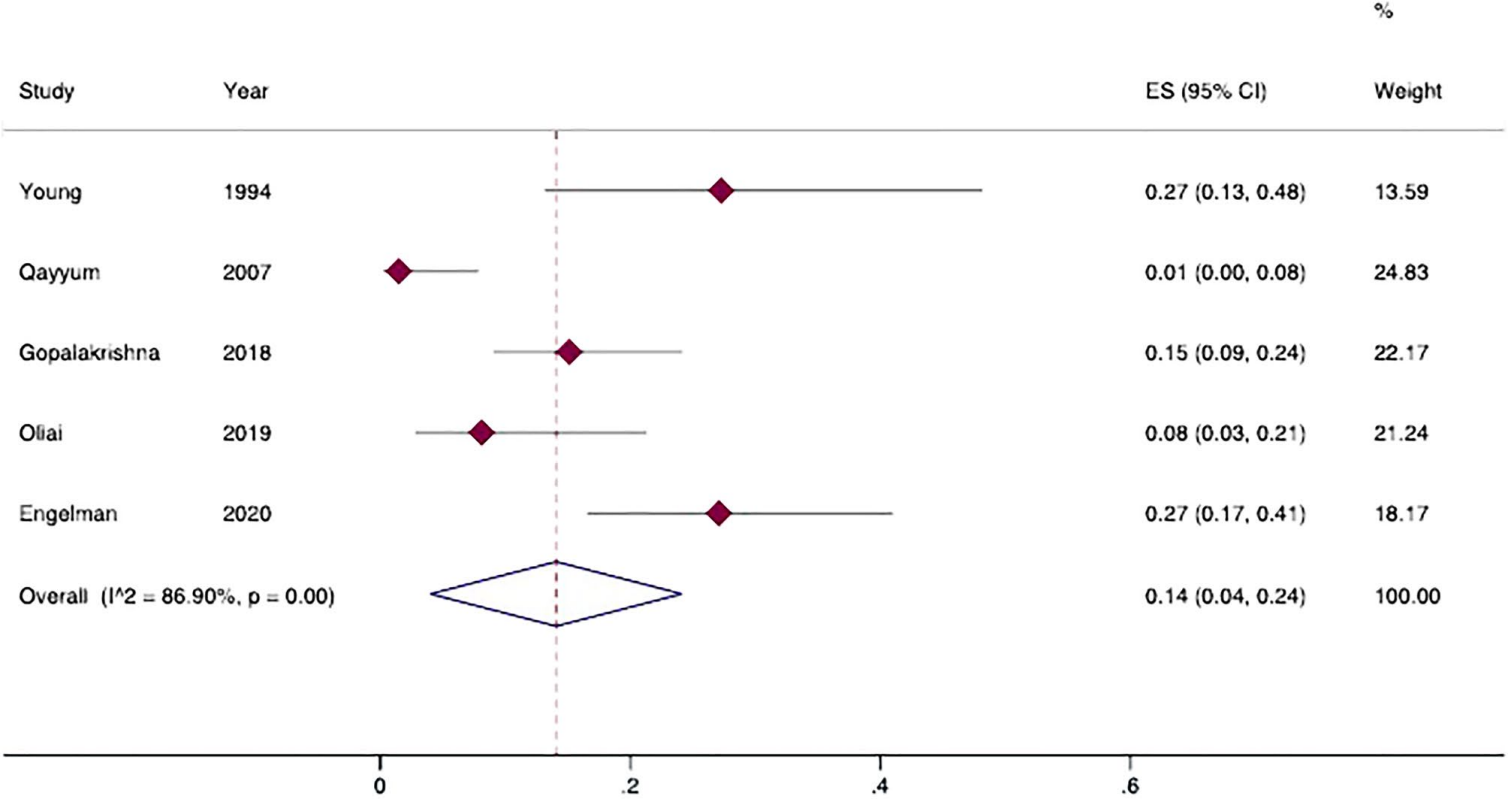
Prevalence of splenomegaly in patients with pseudocirrhosis. Forest plot of overall pooled prevalence of splenomegaly (random effect model); data from observational studies. 95%CI: 95% Confidence Intervals; ES: effect size; I^2^: heterogeneity.

**Figure 4:**
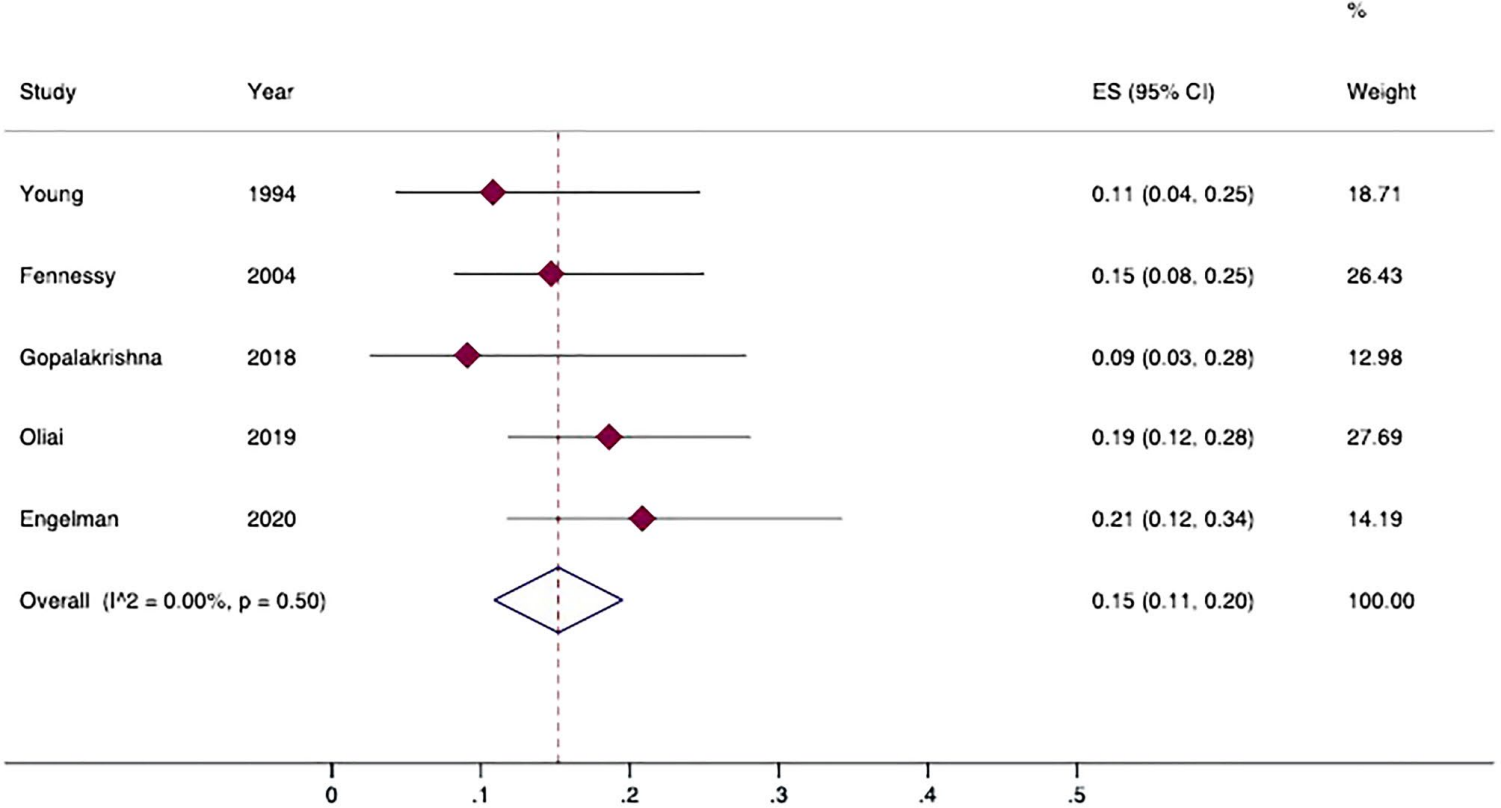
Prevalence of varices in patients with pseudocirrhosis. Forest plot of overall pooled prevalence of varices (random effect model); data from observational studies. 95%CI: 95% Confidence Intervals; ES: effect size; I^2^: heterogeneity.

Five observational studies reported data on portal hypertension (N = 261 patients)^1,7,12,20,21^. The pooled prevalence of ascites, splenomegaly and varices are shown in Figs. 2, 3, 4. Proportion meta-analysis showed that the pooled prevalence of ascites was 50% (95% IC 23–77%; I^2^ 96%; p < 0.001) whereas splenomegaly was less frequent (pooled prevalence 14%; 95% IC 4–24%; I^2^ 86.9%; p < 0.001). Similarly, the prevalence of varices was 15% (95% IC 11–20%; I^2^ 0.31%; p = 0.40).

Data on portal vein thrombosis were reported only by Engelman et al.^12^. These authors observed portal vein thrombosis in 5 of 48 patients (10.4%). This prevalence was similarly reported in case reports (Table 6).

Only one study by Gopalakrishnan et al. reported data on gastrointestinal bleeding in patients with pseudocirrhosis and breast cancer^20^. This study reported bleeding from the upper gastrointestinal tract in 50% of patients with esophageal varices.

Data on hepatic encephalopathy were reported in 2 studies^12,20^. In particular, Gopalakrishnan et al. observed clinical signs of hepatic encephalopathy in 12 out of 86 patients (13.9%) whereas Engelman et al. reported a prevalence of 22.9%.

### Survival and time to death after pseudocirrhosis diagnosis in patients with metastatic cancers—case report and case series

In our analysis we studied the overall survival (N = 23 articles), the median time from liver metastasis diagnosis to pseudocirrhosis (N = 18 articles) and the median time from pseudocirrhosis to death (N = 26 articles). From pooled analysis, patients reported in case reports and case series had a median overall survival of 31 months (IQR 9–162 months) whereas the median time from liver metastasis detection to pseudocirrhosis diagnosis was 8 months (IQR 2.5–16.5). Median survival from pseudocirrhosis to death was 2 months (IQR 1–7).

Notably, two authors reported a complete resolution of pseudocirrhosis^22,23^. Particularly, Shinoda et al. reported a case of diffuse metastases from gastric cancer with a complete resolution after S-1 and oxaliplatin treatment^23^ whereas Kang et al. observed a complete resolution in a 55-year-old asymptomatic woman with metastatic pancreatic cancer^22^. The patient underwent systemic chemotherapy with gemcitabine and oxaliplatin.

### Survival and time to death after pseudocirrhosis diagnosis in patients with metastatic cancers—observational studies

Data from observational studies are very limited because only very few studies reported survival outcomes. The overall survival and time from the first detection of liver metastasis to pseudocirrhosis was reported in only by one study (69 months and 18 months respectively; N = 37 patients)^1^.

Similarly, data on the median time from pseudocirrhosis to death was reported by 2 studies (3.6 and 8.5 months, respectively)^12,24^.

### Anticancer agents in patients with pseudocirrhosis

**Figure 5:**
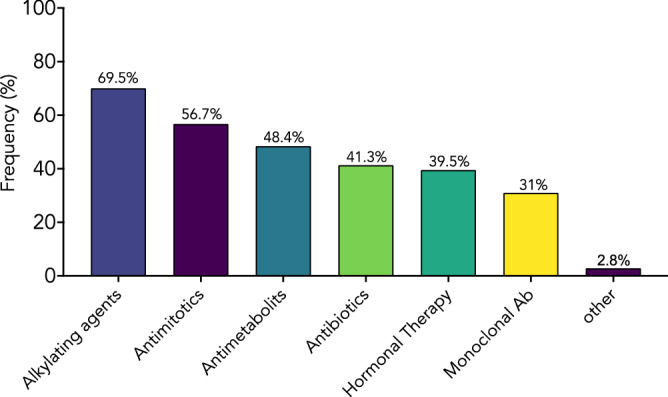
Anticancer drugs in patients with pseudocirrhosis. Anticancer drugs reported in case reports and in case series. Values are expressed as frequencies.

**Figure 6:**
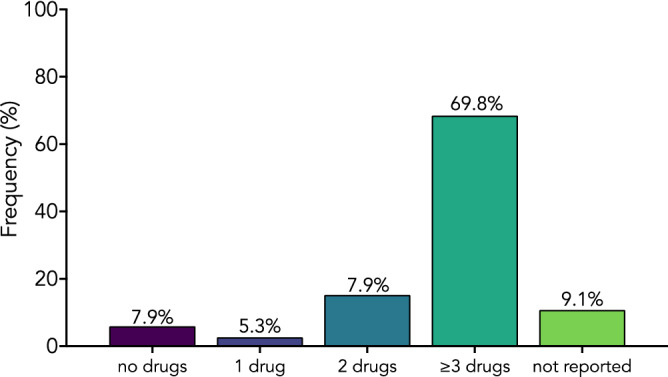
Number of anticancer drugs used in patients in patients with pseudocirrhosis. Data from case reports and case series. Values are expressed as frequencies.

Data on anticancer drugs were available only for case reports and case series. Pooled analysis showed that alkylating agents and antimitotics were the most common class of anticancer drugs used in patients with pseudocirrhosis (69.5% and 56.7% respectively) followed by metabolites (48.4%) (Fig. 5). Notably, about 70% of patients had been given three or more anticancer drugs (Fig. 6).

### Sensitivity analysis

Because of high between-study heterogeneity in meta-analysis of ascites and splenomegaly proportions, we performed a leave-one-out sensitivity analysis (Supplementary Table S4). This strategy did not reduced heterogeneity in analysis addressing the prevalence of ascites, whereas it marginally reduced the heterogeneity when data on prevalence of splenomegaly were considered. Particularly, in this last meta-analysis, the omission of study by Qayyum et al.^21^ changed I^2^ from 86.9 to 60.1%, whereas the leave-one-out method did not affect the heterogeneity when any other study was excluded.

## Discussion

Pseudocirrhosis is a clinical and radiological entity characterized by morphological changes typically observed in patients with liver cirrhosis, such as capsular retraction, decreased hepatic volume with the enlargement of the caudate lobe in patients without a history of chronic liver disease^25^. As for virus- or metabolic-related liver cirrhosis, pseudocirrhosis may associate with portal hypertension and complications of portal hypertension such as gastrointestinal bleeding. Prospective studies on the risk factors and incidence of pseudocirrhosis have not been conducted so far and only small retrospective studies have been published^1,7,12,19,21,24^. Most data are reported in case reports or case series, therefore, very few data are currently available on the prevalence, clinical manifestations and prognosis of patients with pseudocirrhosis.

Concerning prevalence, available data are very different according to the study population. Oliai et al*.* studied a cohort of 199 patients with metastatic breast cancer and the prevalence of pseudocirrhosis was 19%^1^. Qayyum et al. retrospectively analyzed 91 patients with breast cancer and liver metastases who received chemotherapy and underwent multiple CT scan. 68 out of 91 patients (75%) developed hepatic contour abnormalities during a follow-up period of 15 months^21^. Finally, abdominal CT scans of 200 patients with breast cancer were reviewed by Fennessy et al.; 58 patients had liver metastases and 50% of them had hepatic capsular retraction^19^. We performed a systematic review and meta-analysis to address the current knowledge on pseudocirrhosis, its clinical manifestations and impact on survival. Our data have shown that most part of patients had hormone receptor-positive breast cancer, particularly invasive ductal carcinoma. Breast cancer is the most common malignant disease diagnosed in women and nearly 40% of patients with invasive breast cancer have metastases at initial presentation^26^. Liver involvement in metastatic breast cancer is common, however it can be secondary to both metastatic spread and systemic treatment with chemotherapeutic agents^26^. Why this phenomenon is more common for breast cancer remains unclear; however due to the histologic findings, two mechanisms can be potentially involved in pseudocirrhosis development: metastatic liver infiltration associated with significant desmoplastic response and hepatic response to livery injury after treatment with chemotherapeutic agents^8^. The first hypothesis is based on data published by some authors who reported an extensive desmoplastic response in cancer patients with hepatic massive tumor infiltration^8,11^. However, it is already known that chemotherapy-induced livery injury can result in nodular regenerative hyperplasia or hepatic capsular retraction in a decrease in liver lesions or scar formation after parenchymal liver damage. Sonnenblick et al. reported 5 cases of patients who developed pseudocirrhosis after chemotherapy and reduction of hepatic lesion size^10^. Similarly, Young et al. observed a diffuse or focal capsular retraction in patients with pseudocirrhosis. Liver histology confirmed a nodular regenerative hyperplasia, a transformation of normal liver parenchyma into hyperplastic regenerative nodules without bridging fibrosis^7,25^. Concordantly, we found that most common histological findings in patients with pseudocirrhosis are nodular regenerative hyperplasia, diffuse infiltration of tumor cells and extensive stromal fibrosis with compression of the vasculature^26^. Multifocal retraction of the liver capsule and enlargement of the caudate lobe can be observed in this setting^27^.

Several anticancer drugs have been associated with vascular disorders of the liver, such as sinusoidal obstruction syndrome, a condition characterized by areas of dilated sinusoids with congestion associated with liver cell plate atrophy and nodular regenerative hyperplasia^28^. Oxaliplatin, paclitaxel, capecitabine and doxorubicin are well-known causative agents for developing of nodular regenerative hyperplasia^25,29,30^. Our study showed that the most common anticancer drugs associated with pseudocirrhosis development are alkylating agents and antimitotics. Notably, nearly 70% of patients with pseudocirrhosis included in our analysis received 3 or more anticancer drugs; therefore these results suggest that the long-term toxic liver injury can be involved in the pathogenesis of hepatic cirrhosis-like changes and, concordantly, most patients we selected developed pseudocirrhosis after anticancer treatment. This mechanism seems to be very appealing but it does not explain the few cases of pseudocirrhosis reported in untreated patients and moreover it does not explain the rare possibility of resolution reported by two authors^22,23^ because, as widely known, advanced liver fibrosis, especially when associated with portal hypertension, is a irreversible process. Therefore, a larger number of patients are needed to understand both the role of cancer cell infiltration and anticancer therapy in pseudocirrhosis pathogenesis.

Our analysis showed that portal hypertension is a very common complication in patients with pseudocirrhosis and ascites and esophageal varices were the most common. In our pooled analysis, the prevalence of ascites was 43.4% in case report/case series and 50% in observational studies. We found a prevalence of esophageal varices of 24% in case report/case series, whereas pooled analysis from observational studies reported a prevalence of 15%. In this case, the higher prevalence observed in case reports/case series might be related to the reporting bias occurring in observational studies. In a not-negligible percentage of patients, variceal bleeding occurred (about 14%) suggesting that in some cases the clinical presentation can be challenging. These aspects suggest that a multidisciplinary team approach involving oncologists and hepatologists is mandatory for the optimal management of patients with pseudocirrhosis.

The impact of pseudocirrhosis development on survival is currently not well defined. Data from the literature showed that median survival in patients with metastatic breast cancer ranges from 29 to 38 months^31^. Our data have shown that median time from liver metastasis detection to pseudocirrhosis was 8 months, whereas survival from pseudocirrhosis to death was only 2 months, suggesting that the development of pseudocirrhosis significantly impact on the clinical course of metastatic disease.

Interestingly, our literature search reported two cases of complete recovery. The first reported in a 55-year-old patient with pancreatic cancer^22^ who developed lobular hepatic contour and capsular retraction 6 months after starting gemcitabine and oxaliplatin and a disappearance of pseudocirrhosis 14 weeks after treatment discontinuation. The authors concluded that early recognition and discontinuation of therapeutic agents can prevent the liver damage and development of portal hypertension. The second one in a patients with ascites and esophageal varices after four cycles of S-1 and oxaliplatin^23^. The patient underwent paracentesis and diuretic treatment and, finally, he underwent two sessions of endoscopic ligation for esophageal varices. Chemotherapy was re-started, however, oxaliplatin was discontinued and 12 months later hepatic nodules disappeared. This experience suggests that not chemotherapy per se but specific regimens could be involved in the development of pseudocirrhosis.

The limitation of our study is the lack of robust literature data because our analysis is based only on case reports, case series and small observational studies, which are per sè more likely to report beneficial effect of interventional treatment than harms^32^. Therefore, more robust data from larger cohorts of patients are required before definite conclusions. Moreover, criteria for diagnosis reported by different authors are not identical and this could have impacted on the prevalence of the disease. The lack of more detailed data regarding the anticancer therapy in population reported in observational studies, which are clearly larger than that reported in case series but not so well characterized from a therapeutical perspective, may have limited our knowledge on the effect of different classes of anticancer drugs on the risk of pseudocirrhosis development. Finally, the lack of data on dose hasn’t made it possible to investigate the correlation between the dose and the risk of drug-associated pseudocirrhosis. Future studies are needed to address several unmet clinical needs such as understanding the role of dosage of anticancer drugs in the pathogenesis of pseudocirrhosis and why pseudocirrhosis associates strongly with breast cancer even if liver is one of the most common sites for cancer metastasis from different primary tumors.

Our manuscript is the last updated analysis of literature and the first systematic review and meta-analysis addressing this topic.

Finally, pseudocirrhosis is a complication of metastatic cancers and it occurs manly in patients with breast cancers. The development of pseudocirrhosis has a significant and negative impact on survival and influences clinical management strategies in patients with cancer. Specific criteria for diagnosis and guidelines for the clinical management are required to improve the quality of clinical practice.

## Supplementary Information

Supplementary Tables.

Supplementary Information.

## Data Availability

All data generated or analyzed during this study are included in this published article.
