# Supplementary material for: Pseudocirrhosis and portal hypertension in patients with metastatic cancers: a systematic review and meta-analysis
Source: Sci Rep. 2022 Nov 18;12:19865. doi: 10.1038/s41598-022-24241-2 (PMC9674682; doi:10.1038/s41598-022-24241-2)
Supplement: Supplementary file 1 — Supplementary Tables. [file 41598_2022_24241_MOESM1_ESM.docx]

**Supplementary Information**

**Pseudocirrhosis and portal hypertension in patients with metastatic cancers: A systematic review and meta-analysis**

Rosanna Villani*^1^, MD PhD

Francesca Di Cosimo^1^, MD

Moris Sangineto^1^, MD

Antonino Davide Romano^1^, MD PhD

Gaetano Serviddio^1^, MD

^1^ Liver Unit, C.U.R.E. (University Centre for Liver Disease Research and Treatment), Department of Medical and Surgical Sciences, University of Foggia, Foggia, Italy.

***Corresponding author:** Rosanna Villani, C.U.R.E. (University Centre for Liver Disease Research and Treatment), Liver Unit, Department of Medical and Surgical Sciences, University of Foggia, Viale Pinto 1, 71122 Foggia, Italy. E-mail: rosanna.villani@unifg.it

**Supplementary Table S1**: **Assessment of quality of case reports according to Murad’s checklist.**

| **Author** | **Selection** | **Ascertainment** | | **Reporting** | | | | **Causality** | **Overall** |
| --- | --- | --- | --- | --- | --- | --- | --- | --- | --- |
|  |  | **Exposure** | **Outcome** | **Alternative**  **Causes** | **Challenge/**  **Rechallenge** | **Dose/response**  **Effect** | **Follow-up** |  |  |
| *Adler et al.* ^33^  **2019** |  | * | * | * |  |  | * |  | 4 |
| *Battisti et. al.* ^34^  **2014** |  | * | * |  |  |  | * |  | 3 |
| *Borja et al.* ^35^  **1975** |  | * | * | * |  |  | * |  | 4 |
| *Busni et al*. ^2^  **1924** |  | * | * | * |  |  | * |  | 4 |
| *Cerny et al.* ^36^  **1992** |  | * | * | * |  |  | * |  | 4 |
| *Cervoni et al.* ^37^  **2008** | * | * | * | * |  |  | * |  | 5 |
| *Chandrakan et al.* ^38^  **2005** |  | * | * |  |  |  |  |  | 2 |
| *Chin et al.* ^39^  **1987** |  | * |  | * |  |  |  |  | 2 |
| *Deprez et al.* ^40^  **2013** |  | * |  |  |  |  | * |  | 2 |
| *Eidenschink et al*. ^41^  **2018** |  | * | * | * |  |  | * |  | 4 |
| *Finocchi et al.* ^42^  **2019** |  | * | * | * |  |  | * |  | 4 |
| *Fournier et al.* ^43^  **2010** |  | * | * | * |  |  |  |  | 3 |
| *Graber et al.* ^3^  **2010** |  | * | * | * |  |  | * |  | 4 |
| *Harry et al.* ^44^  **2012** | * | * | * | * |  |  | * |  | 5 |
| *Hidalgo-Blanco et al.* ^45^  **2017** |  | * |  | * |  |  |  |  | 2 |
| *Honma et al.* ^46^  **1987** |  | * | * | * |  |  | * |  | 4 |
| *Jungst et al*. ^47^  **2013** |  | * | * | * |  |  | * |  | 4 |
| *Kang et al.* ^22^  **2008** |  | * | * |  |  |  | * |  | 3 |
| *Kashyap et al.* ^48^  **2018** |  | * |  |  |  |  | * |  | 2 |
| Kears et al. ^49^  **2019** |  | * |  | * |  |  |  |  | 2 |
| *Klinge et al.* ^6^  **1988** |  | * |  |  |  |  |  |  | 1 |
| *Kobashigawa et al.* ^50^  2010 |  | * |  |  |  |  |  |  | 1 |
| *Lee et al.* ^8^  **2014** |  | * | * | * |  |  | * |  | 4 |
| *Leyden et al.* ^51^  **2010** |  | * | * | * |  |  | * |  | 4 |
| *Liu et al.* ^52^  **2011** |  | * |  |  |  |  | * |  | 2 |
| *Marzuk et al.* ^53^  **2018** |  | * | * |  |  |  | * |  | 3 |
| *Maynard et al.* ^54^  **1966** |  | * | * | * |  |  | * |  | 4 |
| *Mitani et al.* ^55^  **2016** |  | * | * | * |  |  | * |  | 4 |
| *Nakajima et al.* ^56^  ***2005*** |  | * | * | * |  |  | * |  | 4 |
| *Ojeda et al.* ^57^  **1977** |  | * |  |  |  |  |  |  | 1 |
| *Patel et al.* ^58^  **2017** |  | * | * | * |  |  | * |  | 4 |
| *Sass et al.* ^9^  **2007** |  | * | * | * |  |  | * |  | 4 |
| *Tambe et al.* ^59^  **2020** |  | * |  |  |  |  |  |  | 1 |
| *Teke et al.* ^60^  **2011** |  | * | * | * |  |  | * |  | 4 |
| *Uhlmann et al.* ^61^  **1996** |  | * |  |  |  |  |  |  | 1 |
| *Wallace et al.* ^62^  **2003** |  | * | * | * |  |  | * |  | 4 |
| *Zanazanian et al.* ^63^  **2018** |  | * |  | * |  |  |  |  | 2 |
| *Zeina et al.* ^64^  **2017** |  | * |  | * |  |  | * |  | 3 |
| *Aoyagi et al.* ^65^  **2018** | * | * | * | * |  |  | * |  | 5 |
| *Jeong et al* ^25^  **2013** | * | * | * | * |  |  | * |  | 5 |
| *Nascimento et al* ^11^  **2001** | * | * | * |  |  |  | * |  | 4 |
| *Gravel et al* ^5^  **1996** | * | * | * | * |  |  | * |  | 5 |
| *Qizilbash et al* ^66^  **1987** |  | * |  |  |  |  |  |  | 1 |
| *Vuppalanchi et al .* ^67^  **2016** |  | * | * | * |  |  | * |  | 4 |
| *Geeroms et al .* ^68^  **2018** | * | * | * |  |  |  | * |  | 4 |
| *Mizuyama et al* . ^69^  **2014** |  | * | * |  |  |  |  |  | 2 |
| *Shirkoda et al* ^70^  **2014** | * | * | * | * |  |  | * |  | 5 |
| *Shinoda et al* ^23^  **2022** |  | * | * | * |  |  | * |  | 4 |
| *Shijubou et al* ^71^  **2021** |  | * | * |  |  |  | * |  | 3 |
| *Nakano et al* ^72^  **2021** |  | * | * | * |  |  | * |  | 4 |
| *Basinger et al* ^73^  **2021** |  | * | * | * |  |  | * |  | 4 |

**Supplementary Table S2: Assessment of quality of case series according to Murad’s checklist.**

| **Author** | **Selection** | **Ascertainment** | | **Reporting** | | | | **Causality** | **Overall** |
| --- | --- | --- | --- | --- | --- | --- | --- | --- | --- |
|  |  | **Exposure** | **Outcome** | **Alternative**  **Causes** | **Challenge/**  **Rechallenge** | **Dose/response**  **Effect** | **Follow-up** |  |  |
| *Adike et al.* ^74^  ***2016*** | * | * | * | * |  |  | * |  | 5 |
| *Sonnenblick et al.* ^10^  ***2011*** | * | * | * | * |  |  | * |  | 5 |
| *Alberti et al.* ^4^  ***2015*** | * | * | * | * |  |  | * |  | 5 |
| *Gomez Raposo et al.* ^75^  ***2007*** | * | * |  | * |  |  |  |  | 3 |
| *Shreve et al* ^76^  ***2022*** | * | * | * |  |  |  | * |  | 4 |

**Supplementary Table S3 Risk of bias assessment and quality of observational studies**

| Study | Selection | Comparability | Outcome | Overall quality |
| --- | --- | --- | --- | --- |
| Oliai et al. 2019^1^ | **** | * | *** | 8 |
| Qayyum et al. 2007^21^ | **** | * | *** | 8 |
| Fennessy et al. 2004^19^ | *** | * | *** | 8 |
| Young et al. 1994^7^ | *** |  | ** | 5 |
| Gopalakrishnan et al. 2018^20^ | *** | * | *** | 7 |
| Engelman et al. 2020^12^ | *** |  | ** | 5 |

**Supplementary Table S4: Sensitivity analysis (Leave-one-out method)**

| Omitted Study | Pooled Effect  (95% CI) | I^2^ | p |
| --- | --- | --- | --- |
| Prevalence of ascites | | | |
| Young et al. 1994^7^ | 0.49 (0.18-0.81) | 97.05% | <0.001 |
| Qayyum et al. 2007^21^ | 0.60 (0.36-0.84) | 92.29% | <0.001 |
| Gopalakrishnan et al. 2018^20^ | 0.54 (0.18-0.91) | 97.02% | <0.001 |
| Oliai et al 2019^1^ | 0.46 (0.15-0.77) | 96.70% | <0.001 |
| Engelman et al. 2020^12^ | 0.42 (0.19-0.65) | 92.92% | <0.001 |
| Prevalence of splenomegaly | | | |
| Young et al. 1994^7^ | 0.12 (0.02-0.22) | 87.95% | <0.001 |
| Qayyum et al. 2007^21^ | 0.17 (0.09-0.26) | 60.1% | 0.06 |
| Gopalakrishnan et al. 2018^20^ | 0.14 (0.02-0.27) | 86.81% | <0.001 |
| Oliai et al 2019^1^ | 0.16 (0.03-0.29) | 90% | <0.001 |
| Engelman et al. 2020^12^ | 0.11 (0.01-0.20) | 83.45% | <0.001 |
